# Behavioural adjustments in the social associations of a precocial shorebird mediate the costs and benefits of grouping decisions

**DOI:** 10.1111/1365-2656.13679

**Published:** 2022-02-24

**Authors:** Luke R. Wilde, Rose J. Swift, Nathan R. Senner

**Affiliations:** ^1^ Department of Biological Sciences University of South Carolina Columbia SC USA; ^2^ U. S. Geological Survey Northern Prairie Wildlife Research Center Jamestown ND USA

**Keywords:** heterospecific grouping, Hudsonian godwit, *Limosa haemastica*, precocial chick, protective association, spatiotemporal variation

## Abstract

1. Animals weigh multiple costs and benefits when making grouping decisions. The cost‐avoidance grouping framework proposes that group density, information quality and risk affect an individual’s preference for con or heterospecific groups. However, this assumes the cost–benefit balance of a particular grouping is constant spatiotemporally, which may not always be true. Investigating how spatiotemporal context influences grouping choices is therefore key to understanding how animals contend with changing conditions.
2. Changes in body size during development lead to variable conditions for individuals over short time‐scales that can influence their ecological interactions. Hudsonian godwits *Limosa haemastica*, for instance, form a protective nesting association with a major predator of young godwit chicks, colonial short‐billed gulls *Larus brachyrhynchus*. Godwit broods may avoid areas of higher gull densities when chicks are susceptible to gull predation but likely experience higher risk from alternative predators as a result. Associating with conspecifics could allow godwits to buffer these costs but requires enough other broods with whom to group.
3. To determine how age‐dependent predation risk and conspecific density influence godwit grouping behaviours, we first quantified the time‐dependent effects of con‐ and heterospecific interactions on the mortality risk for godwit chicks throughout development. We then determined how godwit density and chick age affected their associations with con‐ and heterospecific.
4. We found that younger godwit chicks' survival improved with closer association with conspecifics, earlier hatch dates and lower gull densities, whereas older chicks survived better with earlier hatch dates, though this effect was less clear. Concomitantly, godwit broods avoided gulls early in development and when godwit densities were high but maintained loose associations with conspecifics throughout development.
5. We identified how individuals can optimally shift with whom they group according to risks that vary spatially and temporally. Investigating the effects of a species' ecological interactions across spatiotemporal contexts in this way can shed light on how animals adjust their associations according to the costs and benefits of each association.

## Graphical abstract

Using precocial shorebird chicks, Hudsonian godwits, and a major chick predator, short‐billed gulls, the authors show the adjustment in species' associations according to changes in stage‐dependent predation risk can be a behavioral method to avoid costs of sometimes dangerous interactions. Updating the framework around animal grouping decisions to account for shifting trade‐offs in species' associations will improve predictions about community structure.

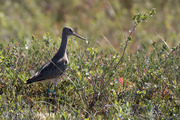

## 1 INTRODUCTION

Animal grouping behaviours affect the structure of ecological communities (Mönkkönen et al., 2007). Groups form around high‐quality habitats or as refuges from predation risk, but the costs and benefits of grouping can vary depending on the environment (Fitzgibbon, 1990; Gil et al., 2017). For instance, grouped individuals have reduced risk of predation and increased foraging efficiency (Gil et al., 2018; Pulliam, 1973), but high group densities can lead to increased competition (Beecham & Farnsworth, 1999; Gil et al., 2017). Additionally, while groups promote shared information about resources and risks (Sridhar et al., 2009), the concentration of individuals can attract predators (Fletcher, 2006). Behaving optimally requires that animals weigh multiple trade‐offs when making grouping decisions (Mönkkönen et al., 2007). Having an accurate cost–benefit assessment is therefore necessary to understand how social animals contend with changing conditions, but monitoring changing conditions across both space and time remains challenging (Elmhagen et al., 2010).

Many species can group with both con‐ and heterospecific (Sridhar & Guttal, 2018), but each group type comes with unique trade‐offs. Conspecific groups are refugia from predation risk and hubs of highly relevant habitat quality information (Fletcher, 2006). However, conspecific groups have higher competition among individuals than do mixed‐species groups (Goodale et al., 2020). Heterospecific groups, on the other hand, can have reduced competition costs while providing a comparatively broader diversity of predator and resource detection capabilities (Sridhar & Guttal, 2018). Nevertheless, the broader range of information can lead to higher rates of misinformation (Magrath et al., 2015). Taken together, each species' population density and the value of the information produced greatly influence which groups are optimal in which environmental context.

**FIGURE 1:**
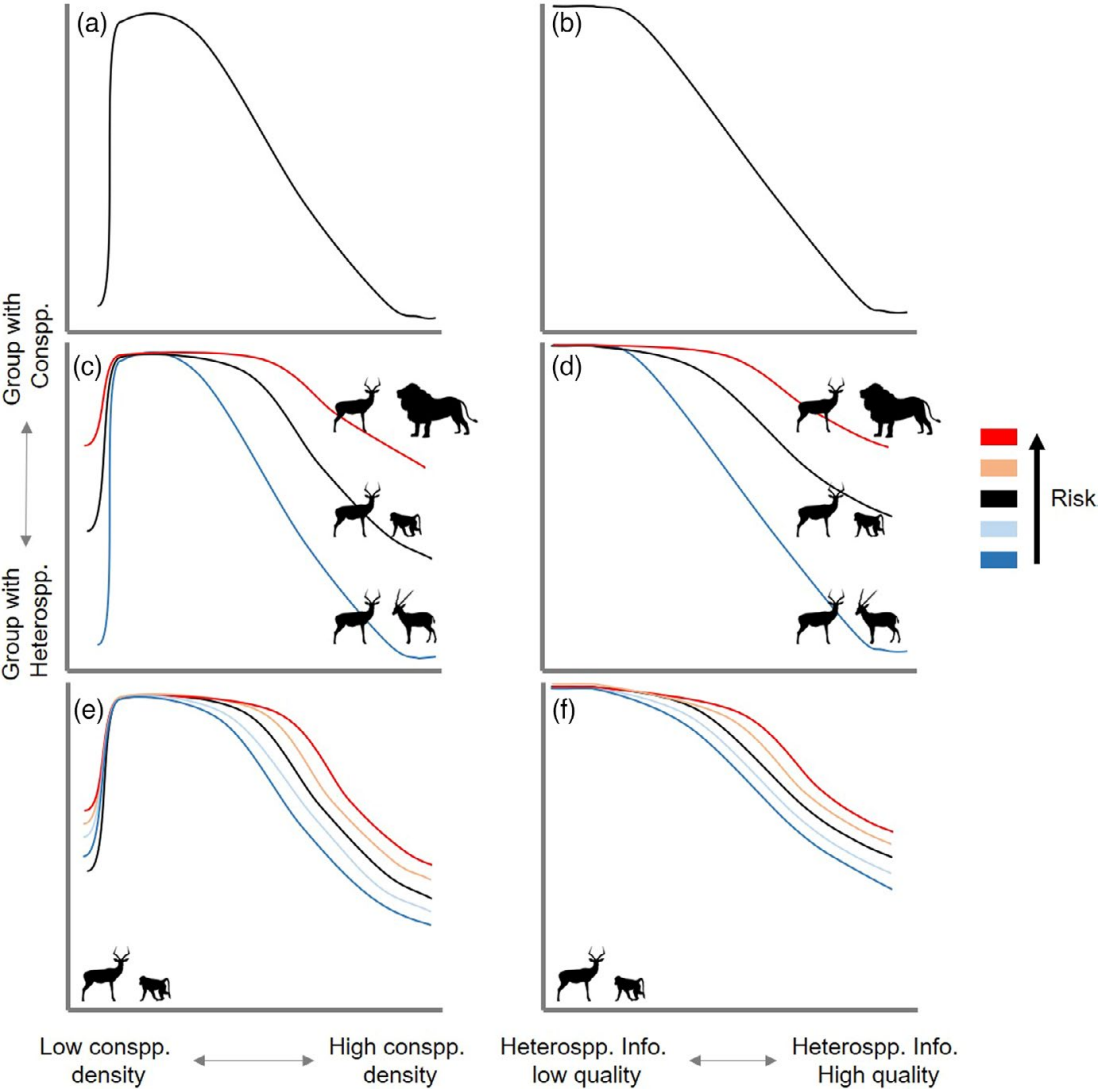
The cost‐avoidance framework supposes that mixed‐species groups are increasingly optimal as (a) conspecific density increase (i.e. competition) or crash (i.e. infrequent encounter) past a cost‐threshold or (b) heterospecifics produce higher quality information relative to conspecifics. Additionally, (c, d) mixed‐species groups are less likely to form when the risk of the heterospecific interaction (blue is low risk, red is high risk) increases. We propose that for interactions with potentially lethal outcomes (e, f), the cost–benefit thresholds affecting selection for mixed‐species groups changes as risk varies (blue is low risk, light blue is moderately low risk, black is moderate risk, yellow is moderately high risk and red is high risk)

Neither con‐ nor heterospecific groups are static, though, and species frequently transition between groups with single or multiple species (Larsen & Grundetjern, 1997). The ‘cost‐avoidance grouping’ framework (Goodale et al., 2020; Trillo et al., 2019) lays out three criteria that should determine when it is optimal for individuals to associate more strongly with hetero‐ than conspecifics: (1) when conspecific groups become too dense or are rarely encountered (Figure 1a; Doligez et al., 2003), (2) when the quality of heterospecific information surpasses that provided by conspecifics (Figure 1b; Meise et al., 2020) and/or (3) when heterospecifics do not pose direct danger (Quinn & Kokorev, 2002; Figure 1c,d). For instance, impala *Aepyceros malampus* frequently group with other ungulates (e.g. Thompson’s gazelles, *Eudorcas thomsonii*) to avoid lions *Panthera leo*, but they also join olive baboon troops *Papio anubis*, a predator of impala calves, when in the presence of other top predators, such as cheetahs *Acinonyx jubatus* (Kiffner et al., 2014).

In its current form, however, the cost‐avoidance grouping framework assumes that the costs and benefits of a particular grouping are constant through time. Interactions between competitors and predator–prey pairs can change with conditions (Cassidy et al., 2020). This is especially true for heterospecific associations in which one species may occasionally pose a threat to the other, such as protective associations and resource provisioning relationships. In these cases, the likelihood of a negative or positive outcome is highly context dependent (Morosinotto et al., 2012; Prugh & Sivy, 2020; Quinn & Kokorev, 2002). For instance, in protective nesting associations, the risk of predation by the ‘protector’ species can depend on the abundance of alternative prey (Larsen & Grundetjern, 1997) or developmental stage of the ‘protected’ species (Haemig, 2001; Morosinotto et al., 2010). By ignoring variable risk within heterospecific interactions across space and time (Willems & Hill, 2009), our current understanding of the factors influencing grouping choices may miss important details about how the cost–benefit threshold can change with spatiotemporal context (Figure 1e,f; Ortiz et al., 2019).

One way to capture interactions across many contexts is by studying a species during periods of rapid change, such as early life development. Changes to the scale and direction of ecological interactions over the course of ontogeny are ubiquitous across taxa (Yang & Rudolf, 2010), but these changes are most clear in size‐structured interactions like predation (Yamaguchi & Kishida, 2016). Shorebirds are a case‐in‐point: shorebird chicks are highly precocial and exhibit rapid growth and mobility changes over short time‐scales (Engström‐Öst & Lehtiniemi, 2004; Królikowska et al., 2016). As a result, the sources and absolute levels of predation risk posed by certain predator classes vary predictably as chicks age (Dreitz, 2009; Schekkerman et al., 2009). Additionally, shorebirds exhibit a wide array of grouping behaviours with con‐ and heterospecifics, but the degree to which these change in response to predation risks has rarely been considered (Dreitz, 2009; Larsen & Grundetjern, 1997). Studying the factors influencing the social behaviour and survival of shorebird chicks could therefore provide important insights into how variable risks affect a species' interactions across environmental contexts.

Hudsonian godwits *Limosa haemastica* (hereafter ‘godwit[s]’) are long‐distance migratory shorebirds that breed across the Nearctic (Walker et al., 2020). The breeding population in southcentral Alaska forms a protective nesting association with colonial short‐billed gulls *Larus brachyrhynchus* (hereafter ‘gull[s]’; previously ‘*Larus canus*’). We have previously shown that godwit nests inside gull colonies have higher survival to hatch, but at the cost of increased predation of young chicks (<5 days; Swift et al., 2018). However, gulls only prey upon young godwit chicks once their own chicks have hatched, meaning that a godwit chick’s hatch date could affect their predation risk from gulls. Furthermore, because gulls are gape‐limited central place foragers (Väänänen et al., 2016), godwits may spatially avoid nesting gulls when their chicks are small and vulnerable to gull predation.

Once outside of gull colonies however, godwit broods (i.e. the avian family unit) are exposed to generalist predators who, together with gulls, account for 87% of chick mortalities (Senner et al., 2017). Godwits may contend with these variable sources and levels of predation risk by remaining near other conspecifics—a behaviour commonly observed in other precocial species (Eadie et al., 1988; Lanctot et al., 1995; Larsen & Moldsvor, 1992)—but the interplay between risk and godwit associations with con‐ and heterospecifics is unclear. Furthermore, the attrition of godwit broods (i.e. declining density later in the breeding season) could hinder their ability to locate conspecifics with whom to group over the course of the season (i.e. Allee effect; Stephens & Sutherland, 1999) and promote the godwit–gull association.

We investigated how variable predation risk and conspecific density shape the associations godwits form with con‐ and heterospecifics. To determine the trade‐offs that godwits encounter throughout the pre‐fledging period, we studied the influence of godwit densities and age‐specific predation risk on godwit chick survival and social behaviour. We first quantified how interactions with con‐ and heterospecifics influenced godwit chick survival. Then, we investigated how the strength of godwits' associations changed with godwit density and chick age. We hypothesized that (1) chick survival relates to con‐ and heterospecific densities, but that younger and older chicks face different predation risks and (2) godwit density and chick age shape the strength of the godwit–gull association. We predicted that young godwit chicks that remained closer to other godwit broods or used areas with lower gull densities would have reduced risk of predation, but that older godwit chicks would reduce risks by avoiding the forest edge. Furthermore, we predicted that godwit broods would avoid gulls when conspecific densities were high or their chicks were young. Testing these hypotheses will help clarify how animals adjust their behaviours to changing conditions and elucidate the effects that population‐level processes can have on animal grouping behaviours.

## 2 MATERIALS AND METHODS

### 2.1 Study area

We monitored the survival and space use of godwit chicks near Beluga River, Alaska (61.21°N, 151.03°W; hereafter, ‘Beluga’) from early‐May to mid‐July (μ = 78 days) during three study periods: 2009–2011, 2014–2016 and 2019. We divided the study region into two plots—North (5.5 km^2^) and South (1.2 km^2^)—separated by 7 km of boreal forest (Figure S1). Both plots contain freshwater ponds, black spruce *Picea mariana* outcroppings and low vegetation (Swift et al., 2017), and each plot hosts a large, centrally located gull colony (North: *x̅* = 66 nests, range = 55–77, South: *x̅* = 51 nests, range = 41–61). Access to field site was granted by Cook Inlet Region Incorporated and the Native Village of Tyonek beforehand.

### 2.2 Nest detection and monitoring

We located godwit nests using behavioural observations and opportunistic encounters while surveying the extent of the plots every 2–3 days (Table S1). We floated godwit eggs to estimate hatch date and monitored nest survival every 2–3 days (Liebezeit et al., 2007). We transitioned to daily visits once eggs showed pipping or starring.

In 2015, 2016 and 2019, we located all gull nests on both plots. Gulls are the most abundant breeder in the area (Swift et al., 2018) and incubating adults are highly visible. Of these nests, we estimated the hatch dates of a subset (*x̅* = 21.3 nests per year, range = 8–45) with repeated nest visits (2015–2016) and egg flotation (2019; Westerskov, 1950). For years when gulls were not fully monitored (2009–2011 and 2014), we approximated nest locations from the average gull nest locations in years with monitoring. We combined all nests in each gull colony from each year and calculated kernel utilization distribution (KUD) isopleth contours as an annual estimate of gull density throughout the plot using the ‘adehabitatHR’ package (Figure S1; Calenge, 2006) within the R programming environment (v.4.0.3; R Development Core Team, 2020).

### 2.3 Godwit chick capture and monitoring

Immediately after hatch, we marked godwit chicks with a unique alpha‐numeric flag and USGS metal band placed on each tibiotarsus. We failed to locate some nests each year (range = 0–4 nests), but opportunistically captured chicks from these broods. We estimated the hatch dates of chicks captured opportunistically using published growth curves (Senner et al., 2017). Because of the conspicuousness of adult godwits during the chick‐rearing phase and the size of the plots, we are confident that we found and monitored all broods each year. All procedures met the ethical standards of the Cornell University (2001‐0051) and University of South Carolina (2449‐101417‐042219) Animal Care and Use Committees, Alaska Department of Fish and Game (20‐024), and USGS (24191).

We randomly selected 1–2 chicks from each brood (range = 7–23 chicks per year) to monitor with radio telemetry. We attached a 0.62 g VHF radio transmitter (Holohil Systems Ltd.) to the skin above the uropygial gland using cyanoacrylate glue. Radios and flags together were <3% of a chick’s mass at hatch and unlikely to affect the survival of shorebird chicks (Senner et al., 2017; Sharpe et al., 2009). We relocated each radioed chick every 1–3 days (Table S1) and recaptured them every 7 days throughout the pre‐fledging period (28–30 days, Walker et al., 2020) to replace the glue under the radios. We estimated each chick’s location with 3–5 azimuths towards the greatest signal strength and converted these to determine each chick’s location within ±10 m using the program LOCATE (v. 3.34, 2011).

When we did not relocate a chick’s radio, we walked concentric circles away from that individual’s last known location. We presumed a chick was dead if we could not detect a signal after three consecutive days. The average life span of our radios was only 21 days (range = 17–33 days; Holohil Systems, 2021), we did not record any radio failures. Furthermore, across all years, 98.6% of relocation attempts were successful (annual range: 0.93–1.00; see *Supporting Information: ‘Treatment of chick relocation records as independent, known fate data’ for details*). Chicks with a missed detection were resighted within 3.2 days (*SD* = 2.0 days; range = 1–7 days) and no chicks considered dead were resighted later (N.R. Senner, University of South Carolina, unpublished data, 2019).

In many cases, when a radioed chick died, other chicks in the brood were still alive (67% of broods). For active broods without a radioed chick, we opportunistically located adult godwits exhibiting clear parental care behaviours (e.g. alarm calling, flights towards observers; Walker et al., 2020) as a rough estimate of the brood’s location (*n* = 335) for use in spatial analyses.

### 2.4 Godwit and gull interaction in space and time

To pinpoint the timing of godwit–gull interactions, we investigated the synchrony between godwit and gull nesting phenologies. We compared the proportion of gull and godwit nests hatched per day of the year using a generalized additive model (GAM) with a beta distribution and logit‐link function with the package ‘gamlss’ (Rigby & Stasinopoulos, 2005).

We tested whether a chick’s nest site constrained their movements and affected the associations godwit chicks formed with con‐ and heterospecifics. Adult godwits partially guide their chicks’ movements, but chicks exhibit independence at hatch (Colwell et al., 2007; Schekkerman & Boele, 2009). Additionally, godwit broods freely traverse other godwits’ nesting territories throughout the pre‐fledging period (R.J. Swift, U.S. Geological Survey, unpublished data, 2019). Nonetheless, a parent’s choice of nest location may influence where chicks move, especially early in development (Schekkerman & Boele, 2009). To test this assumption, we estimated the distance broods moved between consecutive relocations and net‐squared displacement from their nest using the package ‘adehabitatLT’ (Calenge, 2006). Then, we built three separate univariate linear mixed‐effect models (package ‘lme4’; Bates et al., 2020) and tested the effect of nest site on the characteristics of a chick’s subsequent movements by predicting the [1] distance to nearest conspecific neighbour, [2] gull density (i.e. KUD isopleth contour) and [3] distance to the forest edge during the brood stage from the same metric during the nest stage. We included random intercepts for [4] study plot and [5] brood ID, and a random slope term for [6] chick age.

### 2.5 Chick survival

We examined the influence of five factors that we hypothesized could influence chick survival (Table S2). First, at time *t*, we included estimates of [1] nearest conspecific neighbour distance (i.e. the Euclidian distance between tagged chicks) calculated with the package ‘spatstat’ in each plot separately (Baddeley & Turner, 2005) and the [2] godwit brood density per day per plot. Next, we estimated the effect of [3] chick hatch date to account for the survival effects of nesting attempt and phenology (Senner et al., 2017; Wilde et al., 2020). Finally, we tested the effect of heterospecific associations at time *t* with the [4] distance to the forest edge at a chick’s location in each plot separately as a proxy for risk from generalist predators that are typically more abundant nearer the forest edge in boreal regions (Lima, 2009; Robinson et al., 1995; Roos et al., 2018) and [5] relative gull density. Gull nests are spatially clustered within colonies (R.J. Swift, U.S. Geological Survey, unpublished analysis, 2020), we therefore used relative density (i.e. the difference between the observed and predicted KUD of gull density from a spatially explicit linear model throughout both plots) as a predictor variable (Ives & Zhu, 2006; see *Supporting Information: ‘Removal of spatial trends in gull density’ for details*).

We built a mixed‐effect, Cox proportional hazard model (mCPH) to estimate the time‐dependent effects of additive covariates on instantaneous mortality risk at time *t* (‘coxme’; Therneau, 2020). The mixed‐effect Cox proportional hazards model (mCPH) estimates the effect of covariate values with a semi‐parametric function and includes random effects within a frailty model structure (Murray & Sandercock, 2020). Cox models assume that (1) predictor variables have constant effects (i.e. proportionality), (2) survival probability is cumulative, (3) individuals are censored randomly and (4) fates are known. By incorporating random intercepts, mCPH models are robust to deviations from a last assumption, (5) independence among individuals. We first confirmed that multiple chicks from the same brood had independent survival histories (see *Supporting Information: ‘Treatment of chick relocation records as independent, known fate data’ for details; Figure S2*). We suspected differences among broods, plot and study year affected survival. We therefore included a nested random intercept for [6] chick ID within brood ID, and additional random intercepts for [7] study plot and [8] study year. We tested the proportionality assumption of our global model by quantifying the variation in the Schoenfeld residuals over time. Our model showed disproportionality for a subset of predictors (*p*_global_ = 0.01; Table S3; Figure S3). We therefore elected to proceed with a stratified model for ‘young’ (≤14 days) and ‘old’ (>14 days) godwit chicks independently. We chose 14 days as the cut‐point because of our hypothesis that gulls do not prey upon older godwit chicks (see Section 3).

For both models, we estimated the hazard ratio (HR, *e* ^*β*^) of each covariate, where a predictor decreases risk (‘protective’) when an HR < 1 and increases risk (‘hazardous’) when HR > 1. We rescaled each predictor variable by centring and dividing by two standard deviations (Gelman, 2008). We performed model selection using competing models and Akaike’s information criterion scores corrected for small sample sizes (AIC_c_; Burnham & Anderson, 2002) in the package ‘MuMIn’ (Bartoń, 2015). When no single model had a weight (*w*_i_) > 0.90, we used model averaging and report only the conditional averages (Grueber et al., 2011). We made biological interpretations for predictors whose HR had 95% confidence intervals (CI) that did not include one.

While mCPH offers more robust hazard estimation, it does not allow for cumulative survival estimation. We therefore used a simplified model to estimate the effects of predictors in our ‘young’ and ‘old’ mCPH models on cumulative survival. Cumulative survival estimation requires categorical predictors. We used the Levallée–Hidiroglou method from the package ‘stratification’ (Baillargeon & Rivest, 2011) to identify three levels—low, moderate and high—within each predictor variable. The Levallée–Hidiroglou method iteratively estimates within‐group variation to identify the most likely cut‐off boundaries among a specific number of groups specified by a *K*‐means algorithm within the numerical data (Gunning & Horgan, 2007). Finally, we built univariate models to estimate chick survival to fledging with a 95% CI for each level of our predictor variables, with brood ID as the cluster term (package ‘survival’; Therneau, 2015).

### 2.6 Space use overlap between godwit broods and with the gull colony

To determine how godwit broods' spatial associations with con‐ and heterospecifics changed over development, we estimated the amount of space use overlap among godwit broods and between godwit broods and the gull colony. Given our relatively low sample size of brood relocations, we again divided brood locations into ‘young’ and ‘old’. Within each period, we calculated each brood’s 95% KUD as an estimate of brood home range (Calenge, 2006). Because only one godwit brood survived past 14 days in the South plot in all seasons and only one survived overall in 2014, we excluded all South plot broods and the lone 2014 brood on the North plot from this analysis. KUD estimates from similarly small sample sizes can overestimate the kernel area (Fleming & Calabrese, 2017); however, given the relatively short distances godwit chicks travelled between relocations (all ages: *x̅* = 372.80 ± 343.90 m, *n* = 492), we are confident that any bias was minimal.

We calculated the Utilization Distribution Overlap Index (UDOI) to estimate the pairwise amount of space use sharing (i.e. overlap) between con‐ and heterospecifics for young and old broods using each brood’s 95% KUD and the annual 95% KUD of the gull colony (Fieberg & Kochanny, 2005). While similar indices of spatial overlap range from 0 (no overlap) to 1 (complete overlap), UDOI incorporates information on space use and can be >1 (high space use sharing). We calculated four UDOI values for each brood—among broods (conspecific), as well as between broods and the colony (heterospecific)—for young and old chicks and their bootstrapped 95% CIs.

### 2.7 Density and age effects on heterospecific and conspecific spacing

We tested whether the godwit population in Beluga has declined over the study period and, if so, what effect that decline may have had on the association’s godwits formed. We used a linear regression to estimate the change in godwit brood density in our study site across [1] study years. We included [2] Julian day and [3] Julian day^2^ to account for variation in season length.

To determine whether godwit brood density or chick age affected the associations broods formed with con‐ and heterospecifics, we used mixed‐effect generalized additive models (GAMMs) with a Gaussian error‐term and identity link. The Pearson’s correlation between age and density was low (*r* = −0.05, *p* < 0.05). We therefore elected to measure their effects in the same model. We estimated the additive effects of [1] chick age and [2] godwit brood density on both the nearest conspecific neighbour distance and gull density at a chick’s location in separate models, each with [3] brood ID and [4] study year as random intercepts. We included a quadratic term for chick age (age + age^2^) to test for nonlinear effects throughout development.

## 3 RESULTS

We monitored the survival of 128 godwit chicks from 102 broods throughout the study period (2009–2011: *n* = 60; 2014–2016: *n* = 46; 2019: *n* = 22). We located each radio tagged chick an average of 4.3 times (*SD* = 2.71, range = 1–19; *n* = 778), generally 1.07 days apart (*SD* = 0.49 days, range = 1–4 days), and each surviving brood an average of 11 times (SD = 8.89, range = 1–39; *n* = 428), generally 0.6 days apart (SD = 0.82 days, range = 0–2 days). Across all sample years, 24.2% of radioed chicks survived to fledging (i.e. 28 days; Walker et al., 2020), with an average life span of 11.9 days (*SD* = 8.03 days). Fledging success varied across years but, on average, 27% of broods fledged at least one chick (range = 0%–50%).

### 3.1 Godwit and gull interactions in space and time

**FIGURE 2:**
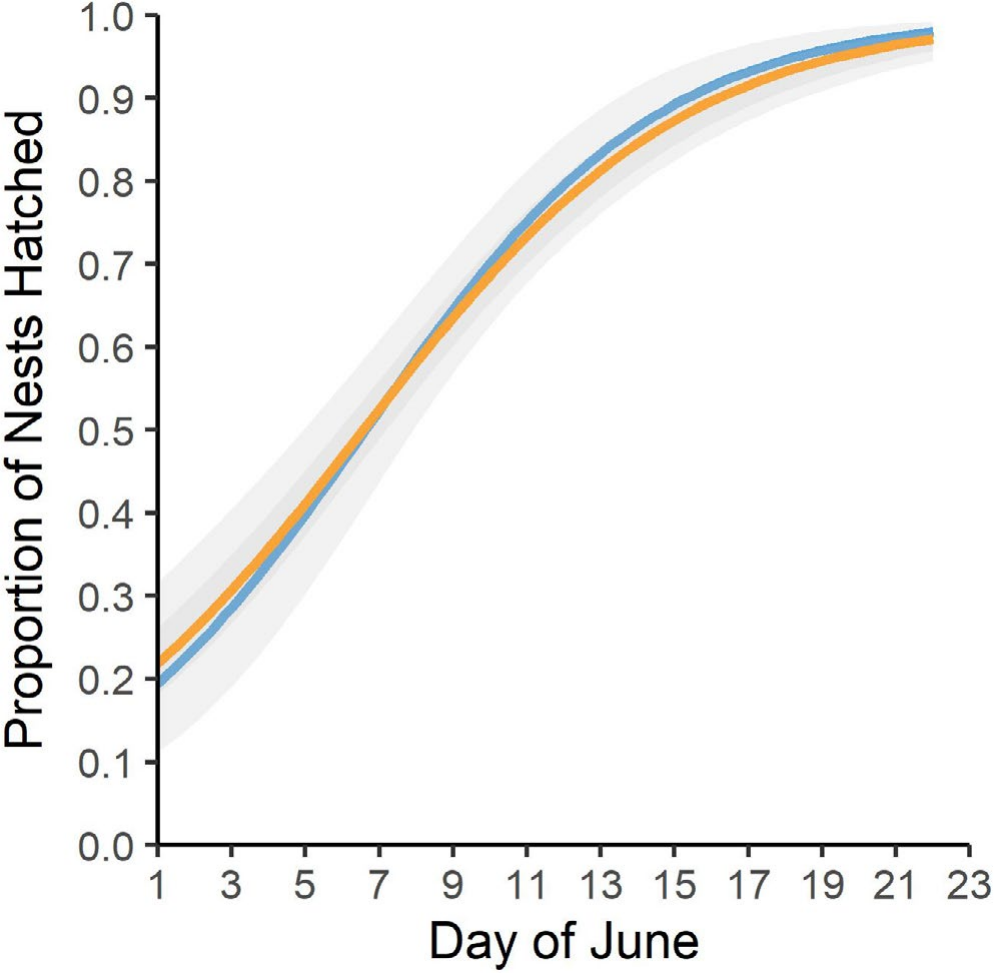
Proportion of Hudsonian godwit (orange) and short‐billed gull (blue) nests hatched by each day in June. Estimates are shown with 95% confidence intervals (grey)

In 2015, 2016 and 2019, we located, on average, 115 gull nests (North: range = 55–77; South: range = 41–61) and estimated the hatch dates of 29 gull nests each season (*n* = 97; range = 10–44). Despite gulls arriving to Beluga several weeks earlier than godwits (Swift et al., 2018), gull and godwit hatch were highly synchronous (difference in hatch dates: *β* = −0.14 ± 0.16 days, 95% CI = −0.45, 0.18; *n* = 119; Figure 2).

Godwit chick movements were not constrained by their nest location. Radio tagged godwit chicks moved on average 373 m between relocations (*SD* = 344 m, range = 36–1311 m; Table S4) regardless of age (*F*_1,364_ = 0.86; *p* > 0.35). Chicks, on average, were found 423 m from their nest (*SD* = 422 m, range = 22–1445 m). Nest site characteristics did not predict the distance to the nearest conspecific neighbour (*β* = 0.04 ± 0.33 m, 95% CI = −0.60, 0.69, $R_{m}^{2}$ < 0.01, $R_{c}^{2}$ = 0.36; *n* = 749; Figure S4, left), gull density (*β* = 0.07 ± 0.19 KUD, 95% CI = −0.17, 0.36, $R_{m}^{2}$ < 0.01, $R_{c}^{2}$ = 0.77; Figure S4, middle) and distance to the forest edge (*β* = −0.05 ± 0.15 m, 95% CI = −0.26, 0.20, $R_{m}^{2}$ < 0.01, $R_{c}^{2}$ = 0.65; Figure S4, right) of a brood. In each case, the random intercept for brood ID and random slope for chick age had 36–77 times larger influence over a chick’s association with con‐ or heterospecifics than the nest site characteristics.

### 3.2 Chick survival

**FIGURE 3:**
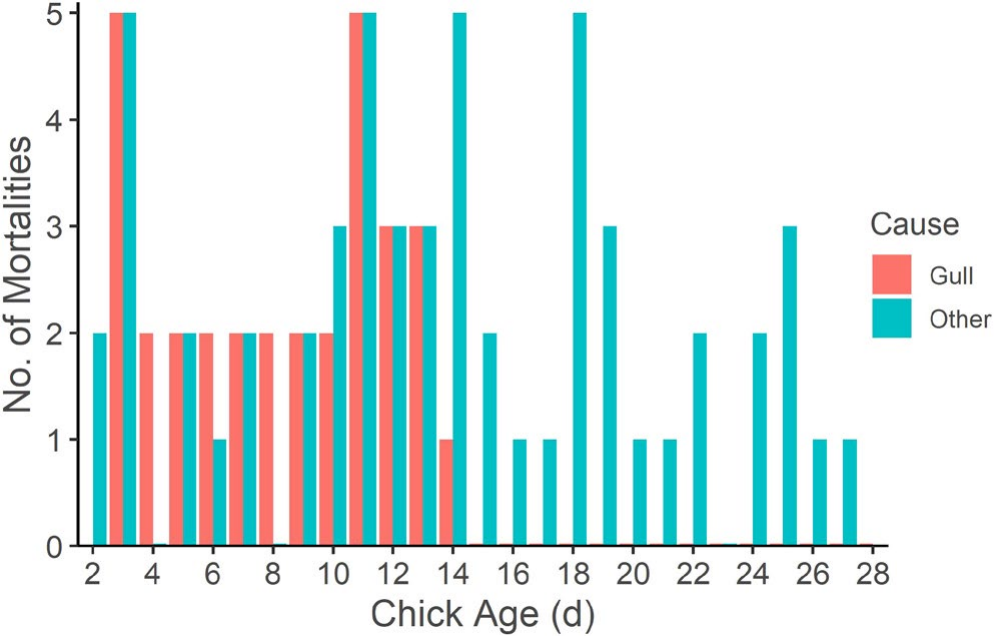
Number of predator‐related Hudsonian godwit chick mortalities directly attributable to short‐billed gulls (red; i.e. ≤25 m of a gull nest) or other predators (blue) by chick age in days

From 2009 to 2019, we recovered the carcasses (*n* = 30) or plucked radios (*n =* 16; i.e. skin and gauze attached) from 52% of all presumed chick mortalities (*n* = 89). Of the instances in which we found a carcass or radio, 73% (*n* = 34) were within the gull colony, and 56.5% (*n* = 26) were on or within 25 m of an active gull nest and therefore likely directly attributable to gull predation. No godwit chicks died from gull predation past 14 days, but 60% of chicks killed by unknown predators died after 14 days (Figure 3). Lastly, locations where we recovered dead godwit chicks had higher gull densities (74 KUD ± 27; range: 10–99) than the locations where we relocated these same chicks while alive (95 KUD ± 23; range: 10–99).

**FIGURE 4:**
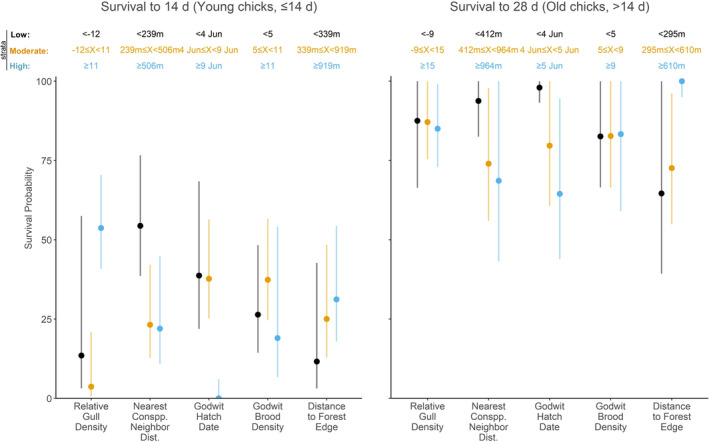
Predicted cumulative survival probability for young (≤14 days; left) and old Hudsonian godwit chicks (>14 days; right) to 14 and 28 days, respectively, from categorical predictor levels. Variables were divided into high (black), moderate (orange) and low (blue) strata using the Levallée–Hidiroglou method. The colour‐coded, cut‐point boundaries are listed above each predictor. Mean estimates (point) are shown with 95% confidence intervals

Our top mCPH model for young chicks included nearest neighbour distance, chick hatch date and gull density (*n* = 490, 77 events, 1,998 iterations; *w*_*i*_ = 0.23; Table S5). Averaged across all candidate models, a young chick’s mortality risk increased by 0.25% with each additional 1 m of distance from its nearest conspecific neighbour and by 7.7% with each additional day after 31 May that it hatched, while it decreased by 1.5% with each 1% increase in gull density relative to nearby spaces (Table 1). Additionally, young chicks within 239 m of a conspecific had >31% better survival to 14 days than those with neighbours further away, while those hatched before 9 June or outside the gull colony’s 85% KUD contour had 37% and 37%–41% higher survival to 14 days than otherwise, respectively (Figure 4).

**TABLE 1:** Effect of predictor variables from the conditional averages of time‐dependent, mixed‐effect cox proportional hazard models on young (*n* = 490) and old (*n* = 200) godwit chicks. Standardized coefficients for predictor variables are reported for comparison among predictors, while unstandardized coefficients are described in the text. Predictors with hazard ratio confidence intervals that do not include one are considered biologically relevant (bold face)

Young chicks (≤14 days)				
Predictor	Relative hazard (*β*)	*SE*	Hazard ratio (HR)	HR 95% CI
Young chicks (≤14 days)				
Predictor	Relative hazard (*β*)	*SE*	Hazard ratio (HR)	HR 95% CI
**Nearest conspp. Neighbour distance**	**1.364**	**0.315**	**3.931**	**(2.113, 7.309)**
Chick hatch date	0.613	0.349	1.809	(0.934, 3.504
Godwit brood density	0.511	0.298	1.657	(0.930, 2.952)
Distance to the forest edge	0.219	0.268	1.245	(0.736, 2.104)
**Relative gull density** ^a^	**−0.733**	**0.243**	**0.476**	**(0.293, 0.771)**

Old chicks (>14 days)				
Predictor	Relative hazard (*β*)	*SE*	Hazard ratio^2^ (HR)	HR 95% CI
Old chicks (>14 days)				
Predictor	Relative hazard (*β*)	*SE*	Hazard ratio^2^ (HR)	HR 95% CI
Nearest conspp. Neighbour distance	−0.107	0.941	0.899	(0.133, 6.057)
Chick hatch date	5.464	3.173	236.1	(0.470, 118,626.0)
Godwit brood density	−1.805	2.451	0.164	(0.001, 20.057)
Distance to the forest edge	−0.268	1.072	0.765	(0.093, 6.252)
Relative gull density	−0.825	0.973	0.438	(0.069, 2.773)

^a^ Relative KUD values (i.e. observed–predicted), with higher values indicating lower density relative to adjacent locations.

Conversely, our top mCPH for older chicks included distance to the forest edge, chick hatch date and godwit brood density (*n* = 200, 18 events, 22,136 iterations; *w*_*i*_ = 0.27; Table S5). Averaged across all candidate models, no predictor had a consistent effect on older chicks' mortality risk, but chicks hatched after 31 May had 65% increased risk for each additional day after 31 May that they were hatched (Table 1), though this effect was highly variable (see *Supporting Information for details; Figure S5*). Older chicks that were hatched before 5 June had 27%–35% higher survival to fledging than chicks that were hatched later (Figure 4).

Finally, while brood mates and chicks from neighbouring broods likely experienced similar risks, chicks from the same brood that both failed to fledge died, on average, 2 days (*SD* = 2.5 days; range = 0–9 days) and 350 m apart (*SD* = 462.9 m; range = 5–1,410 m; *n* = 12), while chicks from separate broods that died on the same day were 644.3 m apart (*SD* = 475.2 m; range = 2–1,694 m; *n* = 34; see *Supporting Information for details; Figure S6*).

### 3.3 Space use overlap between broods and with the gull colony

During both early and late development, godwit broods occupied similarly sized 95% KUD areas (≤14 days: 1.87 ± 0.23 km^2^; *n* = 44; >14 days: 1.82 ± 0.42 km^2^; *n* = 18; Table S4). Young godwit broods exhibited moderate space use sharing with the gull colony (0.32 ± 0.01, 95% CI = 0.25, 0.40; *n* = 44; Figure S7), but old godwit broods exhibited high space use sharing (0.68 ± 0.04, 95% CI = 0.41, 1.05; *n* = 18). Meanwhile, broods had consistently moderate levels of space use sharing with conspecifics (≤14 days: 0.32 ± 0.08, 95% CI = 0.27, 0.37; *n* = 348; >14 days: 0.24 ± 0.01, 95% CI = 0.17, 0.31; *n* = 58).

### 3.4 Density and age effects on heterospecific and conspecific spacing

The maximum godwit brood density occurred on 12 June (*SD* = 3.31 days) and did not differ among years (*F*_1,5_ = 0.90; *p* = 0.39). However, the number of active broods in Beluga on any given day declined over the course of the study (*β* = −0.52 ± 0.06, 95% CI = −0.39, −0.64, $R_{\mathrm{adj}}^{2}$ = 0.58; *n* = 294).

**FIGURE 5:**
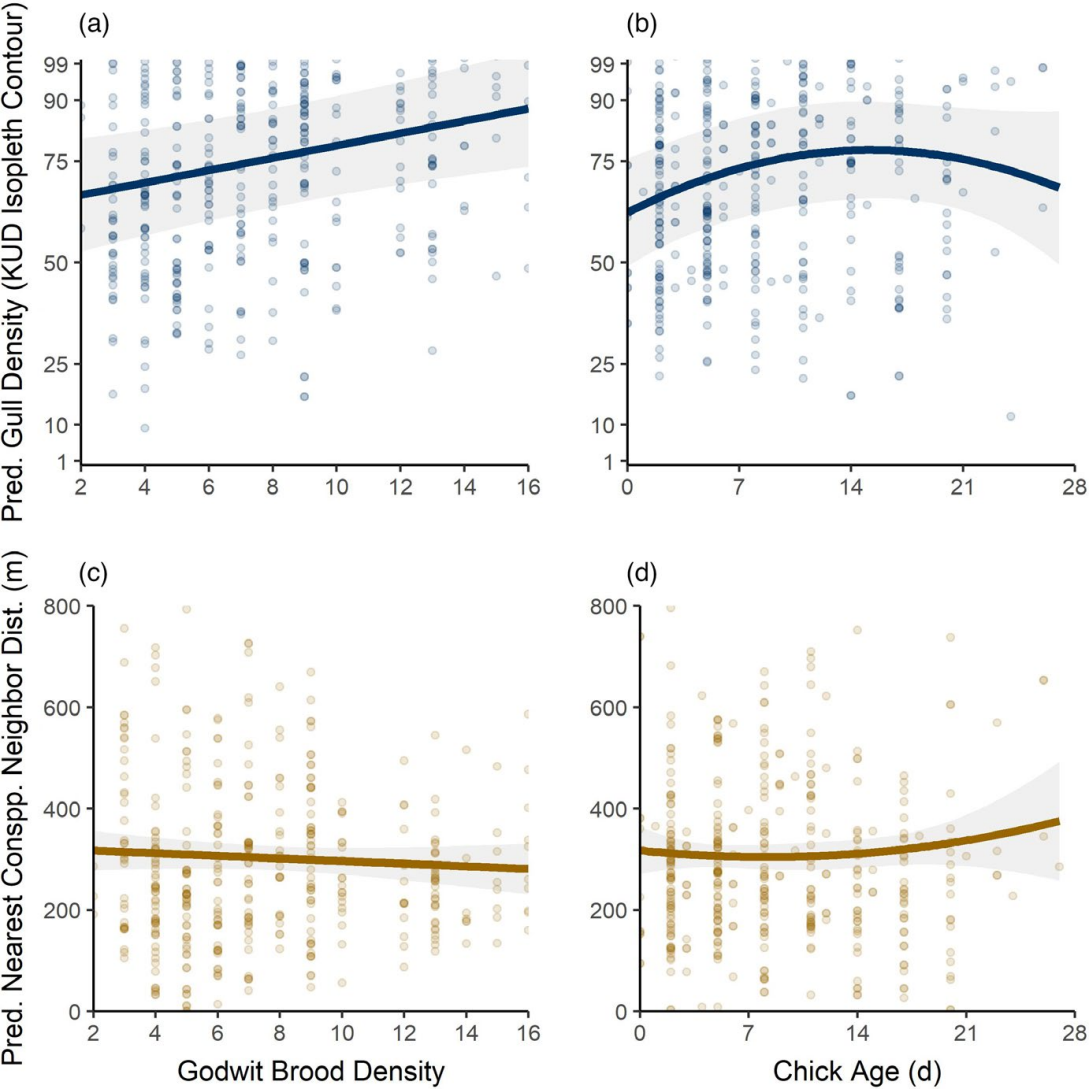
Predicted response of a Hudsonian godwit brood’s experienced short‐billed gull nesting density (a, b; *n* = 425) and nearest conspecific neighbour distance (i.e. Euclidian distance between broods; c, d) to godwit brood density and chick age. Gull densities were estimated from colony KUD isopleth contours, with lower values indicating higher densities. Regression lines from conditional predictions of separate, multivariate GAMMs with random intercepts for brood ID are displayed with 95% confidence intervals

The gull density that godwit broods experienced varied with conspecific density and chick age. Godwit broods used areas with lower gull densities (i.e. lower KUD isopleth contours) when more godwit broods were present (*β* = 1.52 ± 0.52 KUD, 95% CI = 0.50, 2.53, $R_{\mathrm{adj}}^{2}$ = 0.33; *n* = 425; Figure 5a). Concurrently, godwit chick age had a curvilinear effect on the gull colony KUD contour that broods used, whereby broods with young chicks avoided high densities of gulls early but then selected for them once chicks were older (*β* = 2.03 ± 0.73 KUD, 95% CI = 0.61, 3.45; *β* ^2^ = −0.07 ± 0.03 KUD, CI = ‐0.02, −0.13; Figure 5b).

Conversely, the nearest conspecific neighbour distance that godwits maintained was not explained by brood density (*β* = −4.74 ± 3.30 m, 95% CI = −11.22, 1.73; $R_{\mathrm{adj}}^{2}$ = 0.31; *n* = 425; Figure 5c) or chick age (*β* = 3.51 ± 4.57 m, 95% CI = −5.44, 12.47; *β* ^2^ = −0.15 ± 0.20, 95% CI = −0.55, 0.25; Figure 5d). Brood ID explained 39.1% of the variance in the conspecific association, while age and density explained 0.21% and 0.40%, respectively.

## 4 DISCUSSION

A growing consensus suggests that the effects of heterospecific interactions vary in their scale and direction spatiotemporally (Chamberlain et al., 2014; Durant et al., 2005), but this has yet to be integrated into the theoretical framework of group formation (Meise et al., 2020). In support of these earlier studies, we found that young Hudsonian godwit chicks survived better with closer conspecific neighbours, earlier hatch dates and lower short‐billed gull densities, while older chicks' survival showed only a slight improvement with earlier hatch dates. We also found that godwit broods shifted their association with gulls throughout development by avoiding areas with higher gull densities when conspecific densities were high and when chicks were young, but older chicks re‐associated with gulls. Meanwhile, godwit broods maintained consistent associations with conspecifics regardless of brood density or age. Our results provide insights into the grouping behaviours of animals contending with changing environmental conditions and, specifically, potentially dangerous heterospecifics (Morosinotto et al., 2012; Tórrez‐Herrera et al., 2020). Investigating the effects of a species' ecology within a spatiotemporal context can thus shed light on how animals optimally adjust their associations according to the changing costs and benefits of each interaction (Sridhar & Guttal, 2018).

### 4.1 Shifting associations to optimally reduce predation risk

Prey species adjust their space use in response to some predators while tolerating others (Willems & Hill, 2009). Accordingly, we found that godwit broods used areas of lower gull densities early in development and when more conspecifics were available with whom to group. In fact, godwit space use had 112% less overlap with the gull colony when chicks were susceptible to gull predation than when they were not. Species that group with dangerous heterospecifics may thus reduce the strength of the association until the cost–benefit balance favours grouping (Kiffner et al., 2014; Quinn & Kokorev, 2002). A similar mechanism may allow social species to optimally adjust the strength of their associations depending on the trade‐offs presented by specific grouping behaviours (Bicca‐Marques & Garber, 2003; Goodale et al., 2020; Larsen & Grundetjern, 1997).

In contrast to the godwit–gull association, godwits maintained loose associations with conspecifics at all ages and brood densities. Conspecific associations that are favoured regardless of their density can indicate high anti‐predator benefits (Fletcher, 2006; Pulliam, 1973). While the associations we observed between godwit broods were too weak to be considered grouping in the strictest sense, they still likely facilitated the anti‐predator benefits typical of groups. For instance, adults of similar shorebird species are known to broadcast risk information with frequent alarm calls and often haze or physically attack predators in groups involving adults from multiple pairs (Larsen & Moldsvor, 1992). The loose associations godwits formed with other broods may therefore reflect the spatial scale at which information transfers or umbrella protection occurs (Lengyel, 2007; Rocha et al., 2016).

The anti‐predator benefits godwits gain from associating with conspecifics are likely most important when they are outside the gull colony. In other boreal ecosystems, generalist predators are most abundant close to the forest edge (Lima, 2009; Roos et al., 2018). We expected this to be especially true in Beluga because the centrally located gull colonies likely act to repel other predators from the areas furthest away from the forest. Despite generalist predators accounting for ≥27% of all chick mortalities, we found no evidence for survival costs of approaching the forest edge for chicks at any age, suggesting that generalist predation risk may not be as well predicted by distance to the forest edge as in other systems but instead is highly variable throughout our study plots. Given the likely exchange of protection and information among conspecifics, godwits that associate with conspecifics may better navigate the variable and unpredictable risk from generalist predators while avoiding the gull colony.

Ultimately, older chicks reassociated with the gull colony once they were no longer preyed upon by gulls or when conspecifics were at low densities. We hypothesize that the gull colony’s predator detection and deterrence capabilities mean the colony remains a consistent source of risk information and protection for godwits, in addition to what conspecifics alone can provide (Mönkkönen et al., 2007; Vermeer & Devito, 1986). Therefore, while conspecifics provide alternative sources of anti‐predator benefits, grouping with gulls is likely optimal for older chicks that are less vulnerable to gull depredation and as the number of godwit broods dwindles over the course of the breeding season.

### 4.2 Predator–prey synchrony intensifies the risk experienced by later hatched chicks

We found strong directional selection on godwit hatch dates during early and late development, whereby godwit chicks from later nests were less likely to fledge than earlier hatched chicks. Size‐dependent interactions with predators can enact strong selection on reproductive timing (Fuiman, 1994; Start, 2020). In this system, gulls only prey upon young godwit chicks once their own chicks have hatched and require adult provisioning (Swift et al., 2018). Therefore, only godwit chicks that hatch later are likely to experience high predation risk from gulls during the early part of development. Indeed, young godwit chicks that hatched after the median gull hatch date (7 June) experienced 62% higher mortality risk than the earliest godwit nests (31 May). Strong synchrony between predators and their prey is common because predators experience similar abiotic and biotic cues, and even take cues from their prey (Daugaard et al., 2019). As a result, the interplay between predator–prey synchrony and size‐dependent risk may interact and broadly influence predation rates.

Similar survival advantages for earlier hatched chicks have been found in other shorebird studies (McKinnon et al., 2012), but are often attributed to reduced resource availability for later hatched individuals (Saalfeld et al., 2019). Our results suggest that temporally variable predation risk and resource availability may both influence optimal behaviour in godwits, but the degree to which these pressures interact is not well understood. For instance, in this study, godwit chicks were predated by gulls only in the first 14 days of development but were predated by generalist predators at similar rates throughout the pre‐fledging period. Meanwhile, previous observations suggest that the effects of resource availability also change over ontogeny, whereby periods of low resource quality reduce a chick’s survival more strongly as they age and require more energy (Wilde et al., 2020). The fact that neither gull density nor distance to the forest edge predicted the mortality rates of older chicks may therefore suggest that resources, rather than predation, play a stronger role during the later stages of chick development. Ultimately, because the strength of trophic interactions can depend on their timing during development (Durant et al., 2005; Fuiman, 1994), monitoring a species’ interactions with both their predators and prey, as well as how they shift over time, is likely necessary for understanding how animals optimally respond to changing conditions (Damien & Tougeron, 2019; Daugaard et al., 2019).

### 4.3 Conspecific associations in declining species

We found strong evidence of local declines in this population of breeding godwits. Daily densities of godwits on our study plots during the brood‐rearing period have declined by 0.5% per year since 2009. These results are counter to previous surveys of the godwit non‐breeding range that suggested stability in the Alaska breeding population (Andres et al., 2012; Garcia‐Walther et al., 2017). Despite the limited area over which we sampled godwit densities, our findings may be relevant throughout the godwit breeding range considering the density‐dependent behavioural strategies and survival we observed here (see also Swift et al., 2020). For instance, the benefits of conspecific attraction require there be conspecifics with whom to group, which is inherently related to godwit density. Therefore, large‐scale population declines may ultimately intensify the effects of brood attrition over the course of the season, leading to earlier thresholds in the season past which conspecific grouping is infeasible (‘Allee effects’; Stephens & Sutherland, 1999). Godwits as a species have been declining at a rate of ~3.4% annually since 1970 (Rosenberg et al., 2019), meaning that similar effects from reduced conspecific densities may be occurring elsewhere across their range as well. Given the benefits of conspecific associations we observed and the role of conspecific signalling in godwit settlement decisions (Swift et al., 2017), godwits across their range may soon face constraints on their ability to group with conspecifics and be forced to group with potentially dangerous heterospecifics even when it would otherwise not be optimal.

## CONFLICT OF INTEREST

The authors declare no conflict of interest.

## AUTHORS’ CONTRIBUTIONS

R.J.S. and N.R.S. conceived of the study; all authors collected the data; L.R.W. wrote the manuscript and performed the analyses; R.J.S. and N.R.S. provided edits and guidance. All authors gave final approval for publication.

## Supporting information

Supinfo

## Data Availability

Data available from the Dryad Digital Repository https://doi.org/10.5061/dryad.x95x69pfq (Wilde et al., 2022).
