# Supplementary material for: Behavioural adjustments in the social associations of a precocial shorebird mediate the costs and benefits of grouping decisions
Source: J Anim Ecol. 2022 Feb 24;91(4):870–82. doi: 10.1111/1365-2656.13679 (PMC9303437; doi:10.1111/1365-2656.13679)
Supplement: Supplementary file 1 — Supinfo [file JANE-91-870-s001.docx]

**Supporting Information:** **Behavioural adjustments in a precocial shorebird in response to spatiotemporally variable risk mediate costs and benefits of grouping decisions**

Luke R. Wilde^1^*, Rose J. Swift^2^, Nathan R. Senner^1^

*Treatment of chick relocation records as independent, known fate data*

To test support for our treatment of our Hudsonian godwit (*Limosa haemastica*; herafter ‘godwit(s)’) chick survival data as known fate and our use of multiple individuals from the same godwit brood, we checked for imperfect detection of marked individuals and non-independence in survival from individuals within a brood. First, we defined a missed detection as any instance where we failed to locate a chick that was later resighted and confirmed alive. We successfully relocated 98.6% of our radioed chicks (alive or dead) during all attempts, with the lowest annual rate of success being 93% in 2016. Furthermore, of the 128 monitored chicks, only 7% (*n*=9) had one or more missed detections, and rates of missed detections were similar in both North (*n*=4) and South plot (*n*=5). Relocation rates such as this are well above the minimum of 90% detection suggested for limiting bias in survival probabilities from known fate models (DeCesare et al., 2016). Second, we found large variation (median ± SD) in the distance between either the recovered carcass (*n*=23) or last confirmed location (*n*=11) of godwit chicks that died on the same day (635 m ± 475; range: 2–1694 m; Fig. S1), as well as the distance and difference in days godwit chicks from the same brood that both died (350 m ± 463; range: 5–1410 m; 2 d ± 2.5; range: 0–9 d; *n*=12; Fig. S2). This evidence likely justifies our inclusion of multiple chicks from the same brood in our Cox proportional hazards (CPH) and mixed-effect Cox proportional hazards (mCPH) models.

*Removal of spatial trends in gull density*

The short-billed gull (*Larus brachyrhynchus*; herafter ‘gull(s)’) colonies within this system exhibit non-random clustering in nest locations (R.J. Swift, U.S. Geological Survey, unpublished analyses, 2020), a pattern broadly observed in colonial birds (Flemming et al., 2019). Point processes are often subject to spatial autocorrelation, in which another spatial characteristic correlates with the variable of interest. In this case, spatial trends in gull density could correlate with variation in factors that also increase the risk posed to godwit chicks (e.g., distance to the forest edge) or contain variation in the mortality risk posed by clusters of gulls within the colony. We therefore followed methods in Ives and Zhu (2006) to ‘detrend’ our metric of gull density with a spatially explicit linear model performed on the kernel density (KUD) raster of gull nest locations for North and South plot separately in ArcGIS Pro (ESRI 2021). We extracted both the observed and model predicted gull density values at each godwit chick location across either plot and used these to calculate ‘relative gull density’ (i.e., observed - predicted). Rather than comparing gull density to the plot average, relative gull density compares the gull density at a chick’s location to those in the immediate vicinity, thus accounting for unobserved spatial processes that may influence the patterns in gull nest density. We supplanted gull density with relative gull density as a predictor variable in our CPH and mCPH models.

To understand how latent spatial trends in gull density may influence godwit chick mortality risk, we compared the effect size and standard error, on the logit scale, of our global mCPH models for young- and old-chicks with observed and relative gull density. Relative gull density did not substantially affect our conclusions for the drivers of risk in young chicks (Fig. S6), however it did change our conclusions for older chicks. Whereas chick hatch date and distance to the forest edge were both significant predictors of risk for older chicks when observed gull density was included, no predictor had a consistent effect on the risk of older chicks when relative gull density was used. We suspect that this is due to a reduction in the variance inflation among slightly correlated predictors, as is suggested by the increased standard error around the mean effect of godwit density, distance to the forest edge, and hatch date. In the Discussion section of our manuscript, we assert that this suggests that predation may be less important for older chicks than other factors, such as resource availability. In fact, an increasing effect of resource availability with chick age has been observed in this system (Wilde et al., 2020). Therefore, while we still observed shifts in godwit social behavior throughout development, this conclusion agrees with the existing literature on the factors affecting early-life survival in precocial birds.

**References**

DeCesare, N. J., Hebblewhite, M., Lukacs, P. M., & Hervieux, D. (2016). Evaluating sources of censoring and truncation in telemetry-based survival data. *The Journal of Wildlife Management*, *80*(1), 138–148. https://doi.org/10.1002/jwmg.991

Flemming, S. A., Nol, E., Kennedy, L. V., Bédard, A., Giroux, M.-A., & Smith, P. A. (2019). Spatio-temporal responses of predators to hyperabundant geese affect risk of predation for sympatric-nesting species. *PLOS ONE*, *14*(8), e0221727. https://doi.org/10.1371/journal.pone.0221727

Ives, A. R., & Zhu, J. (2006). Statistics For Correlated Data: Phylogenies, Space, And Time. *Ecological Applications*, *16*(1), 20–32. https://doi.org/10.1890/04-0702

Wilde, L. R., Simmons, J. E., Swift, R. J., & Senner, N. R. (2020). The anatomy of a phenological mismatch: Interacting consumer demand and resource characteristics determine the consequences of mismatching. *BioRxiv*, 2020.12.22.423968. https://doi.org/10.1101/2020.12.22.423968

Table S1. Annual summary of Hudsonian godwit nest and chick sampling near Beluga River, Alaska from early-May to mid-July 2009–2019.

| Year | No. godwit  nests found | No. godwit  nests hatched | No.  chicks  hatched | No. radio  tagged  chicks | No. radio  tagged chicks  fledged | Resighting  events per chick  (x̅ (± SD)) | Days chicks  survived |
| --- | --- | --- | --- | --- | --- | --- | --- |
| 2009 | 24 | 19 | 65 | 18 | 5 | 4.0 (± 3) | 11.0 (± 10) |
| 2010 | 24 | 17 | 58 | 19 | 2 | 3.5 (± 3) | 8.8 (± 8) |
| 2011 | 28 | 21 | 72 | 23 | 11 | 4.4 (± 2) | 12.3 (± 7) |
| 2012^*^ | 32 | 20 | - | - | - | - | - |
| 2014 | 11 | 7 | 29 | 7 | 1 | 3.4 (± 2) | 9.3 (± 5) |
| 2015 | 18 | 13 | 45 | 18 | 2 | 3.6 (± 2) | 10.4 (± 7) |
| 2016 | 16 | 10 | 32 | 21 | 2 | 5.5 (± 2) | 15 (± 7) |
| 2019 | 16 | 15 | 48 | 22 | 8 | 5.4 (± 3) | 13.9 (± 9) |
| All years | 169 | 120 | 349 | 128 | 31 | 4.3 (± 3) | 11.9 (± 8) |

Table S2. Annual summary of covariates used in a mixed-effect Cox proportional hazard model estimating the instantaneous risk of death of Hudsonian godwit chicks prior to fledge near Beluga River, Alaska from 2009–2019.

| *Year* | *Godwit hatch date*  *(*$\bar{\boldsymbol{x}}$ *(± SD))* | *Nearest conspecific*  *neighbor*  *distance (m)* | *Relative gull density (observed – predicted KUD)* | *Godwit brood*  *density (daily)* | | *Distance to*  *forest edge (m)* |
| --- | --- | --- | --- | --- | --- | --- |
|  |  |  |  | *North plot* | *South plot* |  |
| 2009 | 4 Jun (± 2.9 d) | 317 (± 186) | 5.62 (± 23.3) | 6.8 (± 1.5) | 2.0 (± 1.2) | 520 (± 176) |
| 2010 | 6 Jun (± 6.4 d) | 472 (± 361) | 0.55 (± 27.8) | 6.4 (± 1.6) | 1.9 (± 1.6) | 478 (± 210) |
| 2011 | 6 Jun (± 4.6 d) | 292 (± 146) | 2.47 (± 27.3) | 12.1 (± 2.5) | 1.5 (± 0.6) | 484 (± 215) |
| 2014 | 7 Jun (± 5.2 d) | 553 (± 285) | -4.38 (± 19.9) | 3.5 (± 0.7) | 1.3 (± 0.9) | 438 (± 221) |
| 2015 | 5 Jun (± 3.1 d) | 320 (± 187) | -11.6 (± 28.3) | 4.9 (± 1.1) | 1.2 (± 1.9) | 402 (± 199) |
| 2016 | 5 Jun (± 2.3 d) | 204 (± 179) | -2.03 (± 24.4) | 4.9 (± 0.7) | 1.6 (± 0.9) | 324 (± 198) |
| 2019 | 5 Jun (± 4.5 d) | 467 (± 417) | 7.45 (± 14.3) | 7.0 (± 2.3) | 2.0 (± 0.7) | 501 (± 258) |
| All years | 5 Jun (± 4.3 d) | 361 (± 298) | 0.53 (± 24.3) | 7.4 (± 3.3) | 1.9 (± 1.3)  (±437) | 458 (± 227) |

Table S3. Shapiro-Wilkes test of proportionality in the Schoenfeld residuals from a mixed-effect, time-to-event Cox proportional hazards model performed on survival data from Hudsonian godwit chicks near Beluga River, Alaska from 2009–2019.

| *Predictor* | *Χ^2^* | *df* | *p-value* |
| --- | --- | --- | --- |
| Nearest conspecific neighbor distance | 0.062 | 1 | 0.803 |
| **Relative gull density** | **4.127** | **1** | **0.042** |
| **Distance to the forest edge** | **9.412** | **1** | **0.002** |
| Chick hatch date | 0.009 | 1 | 0.924 |
| Godwit brood density | 3.487 | 1 | 0.062 |
| **Global** | **15.630** | **5** | **0.008** |

Table S4. Annual spatial summary statistics and monitoring frequency of radio tagged Hudsonian godwit broods in the North plot near Beluga River, Alaska across study years. The single brood from 2014 was excluded due to low sample size.

|  |  |  |  |  | *Young (≤14 days)* | | *Old (>14 days)* | |
| --- | --- | --- | --- | --- | --- | --- | --- | --- |
| *Year* | *Number*  *of broods* | *Loc.*  *per*  *brood*  *(x̄ ± SD)* | *Relocation*  *frequency (days)* | *Distance*  *travelled*  *t+1 (m)* | *KUD^2^*  *area (km^2^)*  *(x̄ ± SE)* | *Number*  *of*  *broods* | *KUD*  *area* | *Number*  *of*  *broods* |
| 2009 | 7 | 6 (± 0.93) | 1.75 (± 1.03) | 391 (± 306) | 1.43 (± 0.50) | 4 | 2.64 (± 1.52) | 2 |
| 2010 | 4 | 7 (± 0.84) | 2.29 (± 1.22) | 326 (± 262) | 1.90 (± 0.74) | 2 | 0.60 (± 0.21) | 2 |
| 2011 | 12 | 7 (± 0.83) | 1.15 (± 0.55) | 324 (± 269) | 1.74 (± 0.42) | 12 | 0.66 (± 0.29) | 4 |
| 2014 | 1 | 4 (± --) | 0.55 (± 0.32) | 225 (± 127) | - | - | - | - |
| 2015 | 12 | 11 (± 0.69) | 0.87 (± 0.52) | 371 (± 284) | 1.65 (± 0.33) | 9 | 1.07 (± 0.25) | 3 |
| 2016 | 4 | 21 (± 0.46) | 0.88 (± 0.26) | 443 (± 423) | 2.30 (± 0.95) | 4 | 2.52 (± 1.39) | 3 |
| 2019 | 9 | 16 (± 0.57) | 1.16 (± 0.27) | 382 (± 423) | 2.29 (± 0.81) | 9 | 3.22 (± 1.55) | 4 |
| All years | 49 | 11 (± 8.89) | 1.07 (± 0.49) | 373 (± 344) | 1.87 (± 0.23) | 44 | 1.82 (± 0.47) | 18 |

Table S5. Model selection table from the stratified, mixed-effect Cox proportional hazards models to estimate the instantaneous risk of death of young (≤14 d) and old (>14 d) Hudsonian godwit chicks prior to fledge from 2009–2019 near Beluga River, Alaska. Model selection was done within the package ‘MuMIn’ on all combinations of standardized predictor variables. Model’s with Δ_AICc_ < 4 and the ‘intercept only’ model are reported, organized by descending Δ_AICc_ value. Plus signs (+) indicate the inclusion within the model.

|  | | | *Young chicks (≤ 14 d)* | | | | | |  | | | | |
| --- | --- | --- | --- | --- | --- | --- | --- | --- | --- | --- | --- | --- | --- |
| *Model no.* | *Nearest conspecific*  *neighbor*  *distance* | *Godwit*  *hatch date* | | *Relative gull*  *density* | *Godwit brood*  *density* | | *Dist. to the*  *forest*  *edge* | | | *df* | *AICc* | *Δ_AICc_* | *Model*  *weight* |
| 29 | + | + | |  | + | |  | | | 28 | 569.4 | 0.00 | 0.225 |
| 31 | + | + | |  | + | | + | | | 28 | 569.8 | 0.36 | 0.188 |
| 30 | + | + | | + | + | |  | | | 37 | 570.0 | 0.55 | 0.171 |
| 32 | + | + | | + | + | | + | | | 36 | 570.1 | 0.73 | 0.156 |
| 22 | + | + | | + |  | |  | | | 36 | 570.2 | 0.77 | 0.153 |
| 24 | + | + | | + |  | | + | | | 36 | 570.9 | 1.49 | 0.107 |
| 1 |  |  | |  |  | |  | | | 39 | 595.3 | 25.8 | 0.000 |
|  | | | *Old chicks (>14 d)* | | | | | |  | | | | |
| *Model no.* | *Nearest conspecific*  *neighbor*  *distance* | *Godwit*  *hatch date* | | *Relative gull*  *density* | | *Godwit brood*  *density* | | *Dist. to the*  *forest*  *edge* | | *df* | *AICc* | *Δ_AICc_* | *Model*  *weight* |
| 15 |  | + | |  | | + | | + | | 7 | 33.6 | 0.00 | 0.271 |
| 7 |  | + | |  | |  | | + | | 6 | 34.8 | 1.16 | 0.152 |
| 31 | + | + | |  | | + | | + | | 8 | 35.3 | 1.61 | 0.122 |
| 30 | + | + | | + | | + | |  | | 8 | 35.3 | 1.64 | 0.120 |
| 13 |  | + | |  | | + | |  | | 6 | 36.1 | 2.46 | 0.079 |
| 16 |  | + | | + | | + | | + | | 8 | 36.2 | 2.57 | 0.075 |
| 32 | + | + | | + | | + | | + | | 9 | 36.5 | 2.88 | 0.064 |
| 29 | + | + | |  | | + | |  | | 7 | 36.7 | 3.01 | 0.060 |
| 14 |  | + | | + | | + | |  | | 7 | 36.8 | 3.14 | 0.057 |
| 1 |  |  | |  | |  | |  | | 12 | 60.7 | 27.1 | 0.000 |


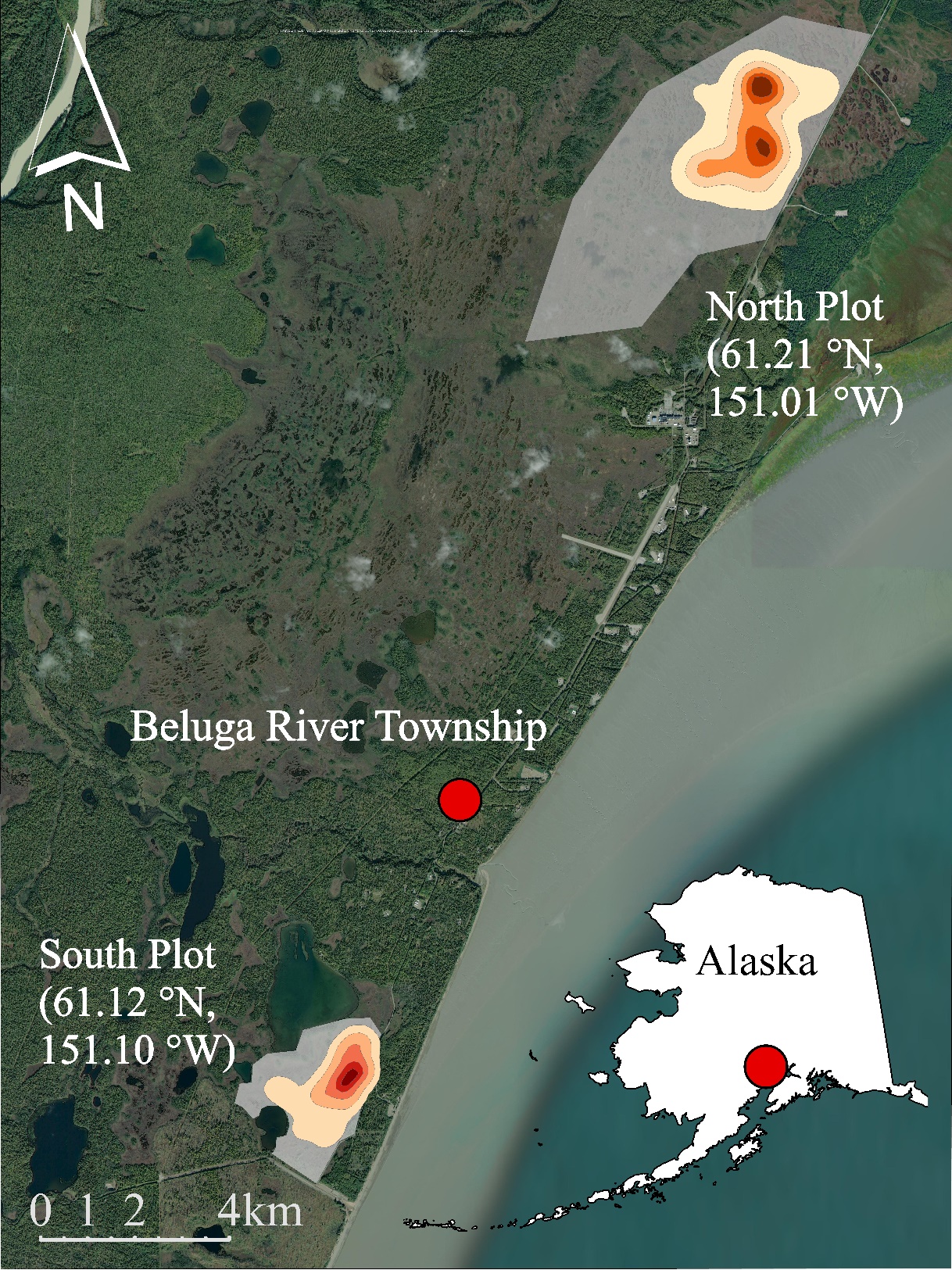


**Figure S1.** Map of the 2009–2019 study area with the North and South plots (grey polygons) depicted, as well as the township of Beluga River, Alaska (red dot). The interannual Kernel Utilization Distribution (KUD) isopleth contours of the North and South short-billed gull colonies are shown as multicolored polygons. KUD isopleths approximate the probability of occurrence for point data; here, increasingly dark red colors indicate higher densities of short-billed gulls. (Inset) Location of study region (red dot) within the Alaska state-boundary. The satellite imagery is the intellectual property of Esri and are used herein under license. Copyright © 2014 Esri and its Licensors. All rights reserved. Additional data sources: U.S. Census Bureau 2018.


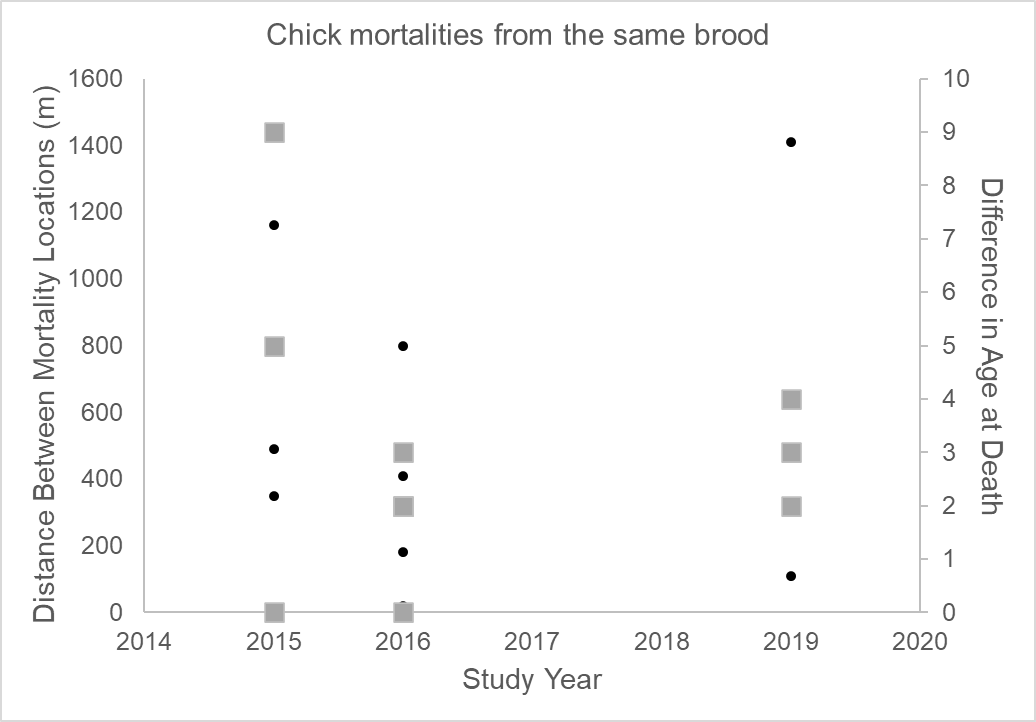


**Figure S2.** Monitoring data from 2009–2019 showing distance (black dots) and difference in days (grey squares) between the death locations and dates, respectively, of Hudsonian godwit chicks from the same brood near Beluga River, Alaska.


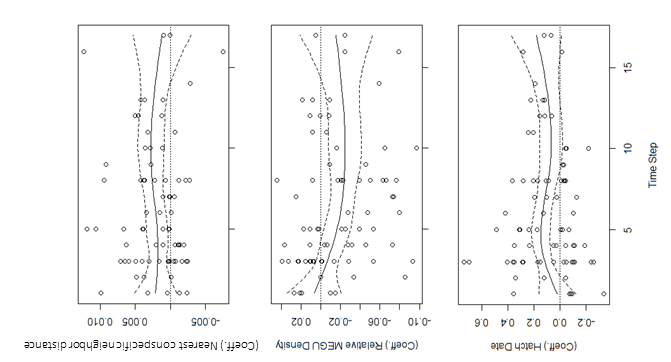


**Figure S3 (cont.)**


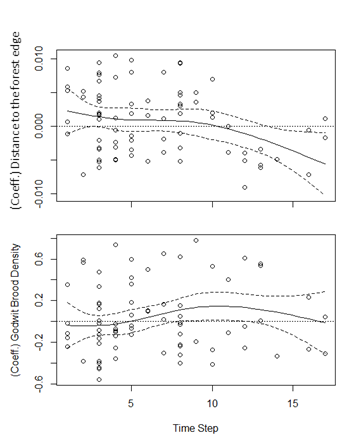


**Figure S3.** Time-specific standardized effects of additive predictors – (in order, top to bottom) [1] nearest conspecific neighbor distance, [2] short-billed gull density, [3] distance to the forest edge, [4] Hudsonian godwit hatch date, and [5] Hudsonian godwit brood density (daily) – across observation intervals in a global mixed-effect Cox proportional hazards model using survival records of Hudsonian godwit chicks near Beluga River, Alaska from 2009–2019. Lack of a trend in the mean coefficient estimate (solid line) is evidence for minimal disproportionality (i.e., constant predictor effects) in the model covariate. Covariates were rescaled to z values by dividing by 2 standard deviations. Time (x-axis) is the sample number per individual (i.e., every 2–3 d).


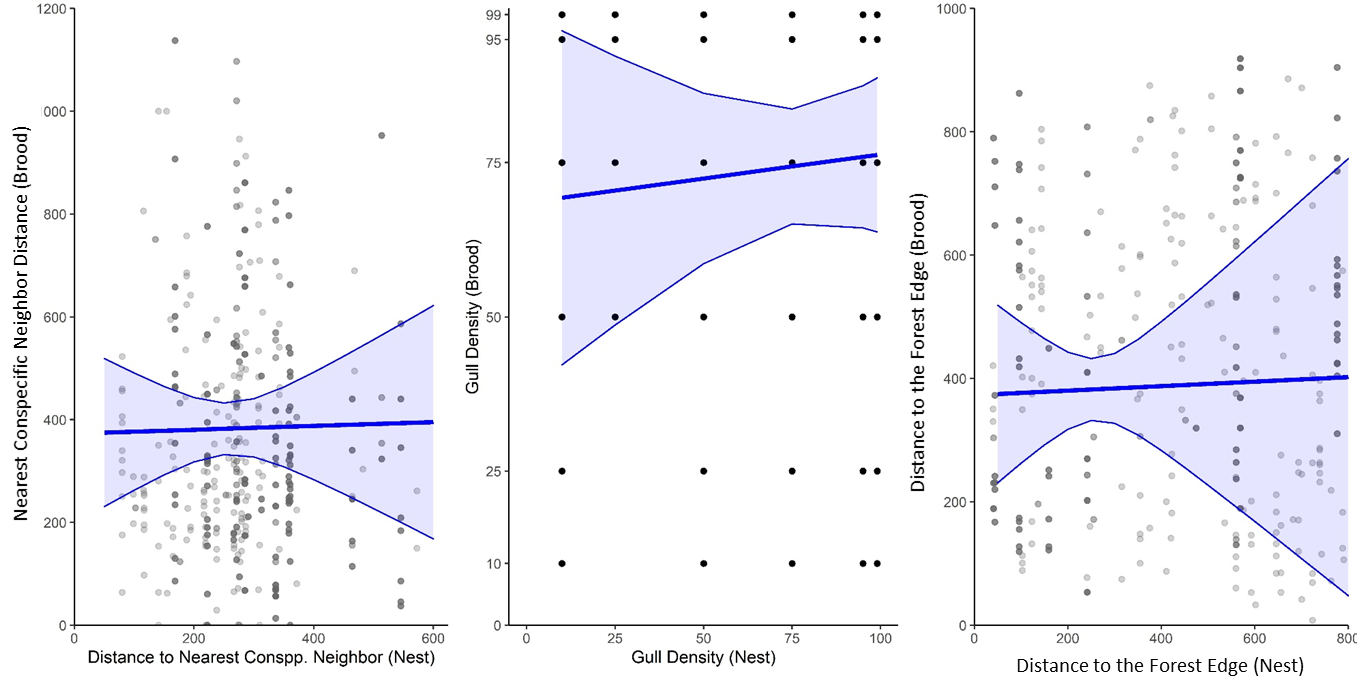


**Figure S4.** Effect of distance to nearest conspecific neighbor (left), short-billed gull density (middle), and distance to the forest edge (right) during the nest phase on the same metrics during the brood stage in Hudsonian godwit chicks, monitored near Beluga River, Alaska from 2009–2019. Predictions from a mixed-effect linear model are shown in dark blue with the 95% CI and include random intercepts for study year and brood ID, and random slopes for chick age.


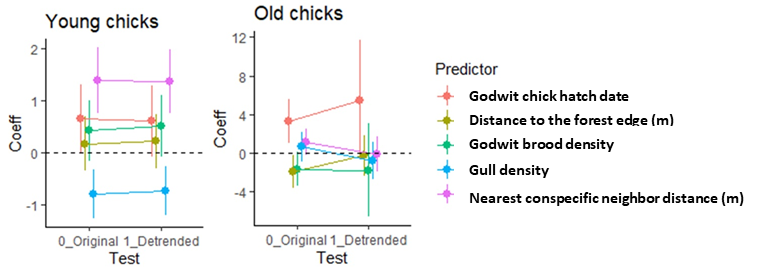


**Figure S5.** Mean effect size (logit scale) and 95% CI of predictor effects in mCPH models of young (left) and old Hudsonian godwit chicks (right) from 2009–2019 near Beluga River, Alaska. ‘0_Original’ signifies the predictor effects using observed gull density while ‘1_Detrended’ represents the predict effects using relative gull density. Colors of lines and points (with error bars) correspond to predictors.


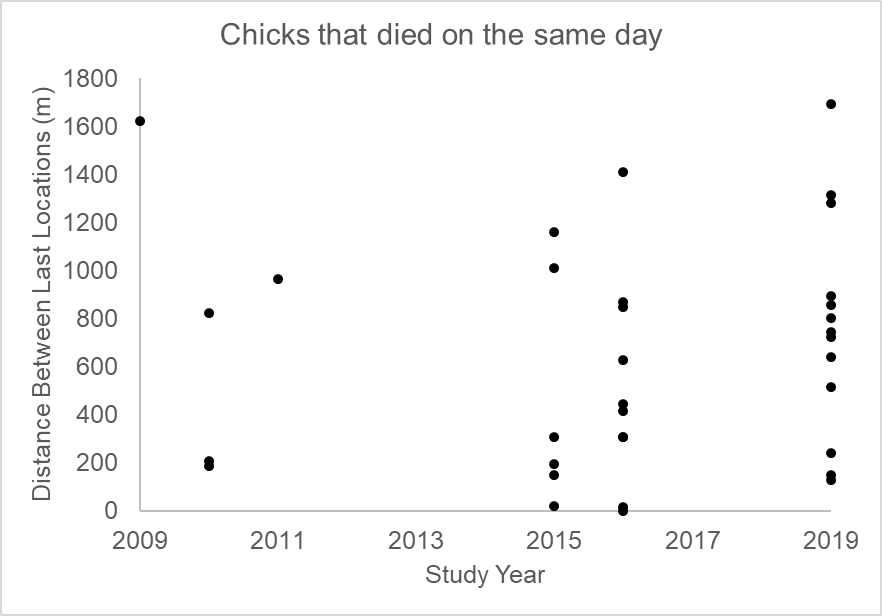


**Figure S6.** Monitoring data from 2009–2019 near Beluga River, Alaska showing straight-line distance (m) between Hudsonian godwit chick mortalities.


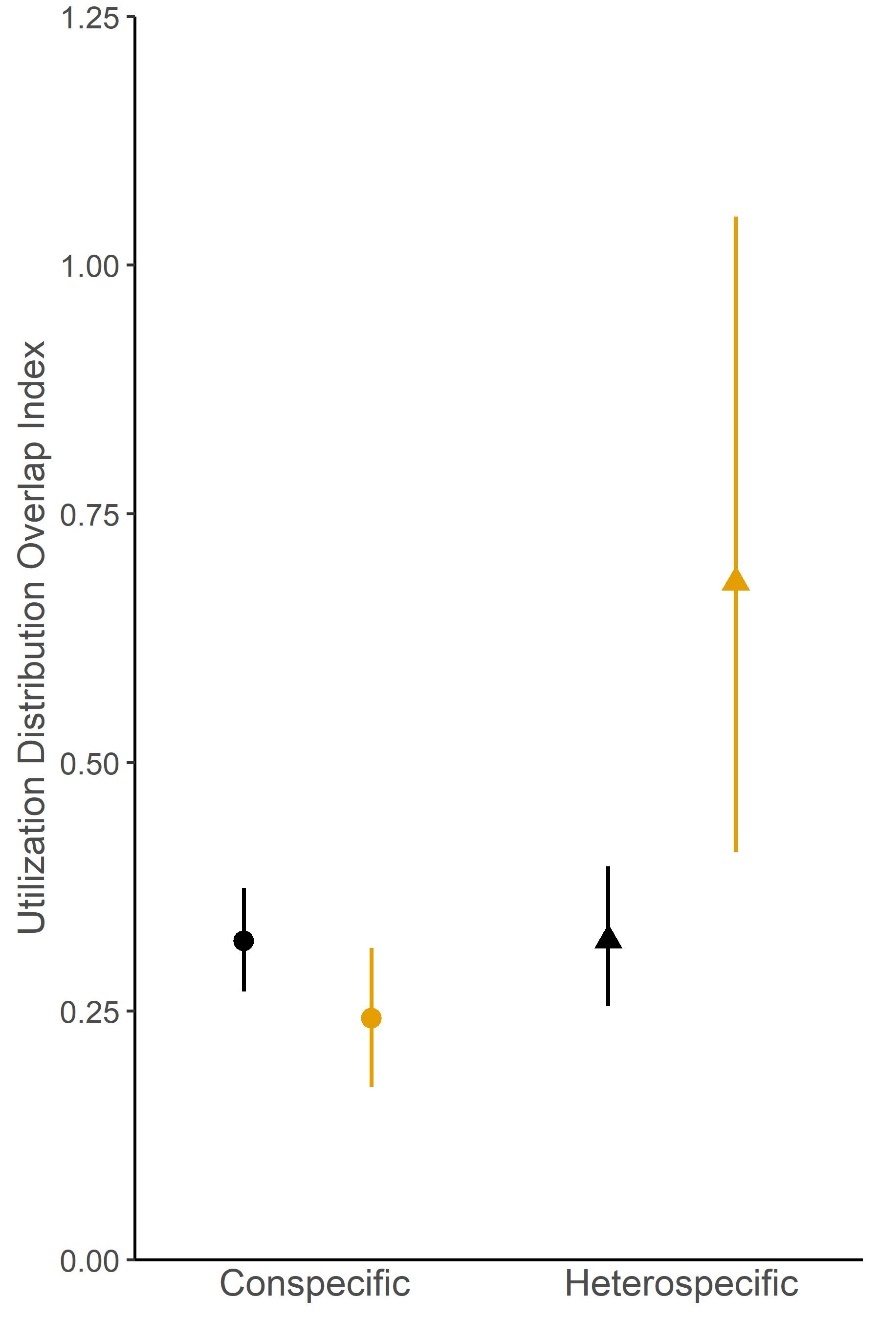


**Figure S7.** Space use sharing between Hudsonian godwit broods (circles; *n* = 342) and with the short-billed gull colony (triangles; *n* = 116) near Beluga River, Alaska monitored from 2009–2019 (except 2012, 2014). Higher utilization distribution overlap index (UDOI) values (y–axis) indicate a higher degree of overlap. Conspecific: UDOI between broods during the first 14 days of the pre–fledge period (‘young’, black; *n*_B–B_=302) and last 14 days (‘old’, yellow; *n*_B–B_=40). Heterospecific: UDOI of broods with the year–specific gull colony during the early (black triangle; *n*_B–C_=80) and late period (yellow triangle; *n*_B–C_=36). Mean estimates of pairwise UDOI are shown with bootstrapped 95% confidence intervals (*n*=100).
